# Tablet-Based Screening of Depressive Symptoms in Quito, Ecuador: Efficiency in Primary Care

**DOI:** 10.1155/2014/845397

**Published:** 2014-02-17

**Authors:** Michelle Grunauer, David Schrock, Eric Fabara, Gabriela Jimenez, Aimee Miller, Zongshan Lai, Amy Kilbourne, Melvin G. McInnis

**Affiliations:** ^1^Universidad San Francisco de Quito, 17-1200-841 Quito, Ecuador; ^2^University of Michigan, Ann Arbor, MI 48109, USA; ^3^University of Michigan Depression Center, 4250 Plymouth Road, Ann Arbor, MI 48109, USA

## Abstract

Depression is a frequent yet overlooked occurrence in primary health care clinics worldwide. Depression and related health screening instruments are available but are rarely used consistently. The availability of technologically based instruments in the assessments offers novel approaches for gathering, storing, and assessing data that includes self-reported symptom severity from the patients themselves as well as clinician recorded information. In a suburban primary health care clinic in Quito, Ecuador, we tested the feasibility and utility of computer tablet-based assessments to evaluate clinic attendees for depression symptoms with the goal of developing effective screening and monitoring tools in the primary care clinics. We assessed individuals using the 9-item Patient Health Questionnaire, the Quick Inventory of Depressive Symptoms-Self-Report, the 12-item General Health Questionnaire, the Clinical Global Impression Severity, and a DSM-IV checklist of symptoms. We found that 20% of individuals had a PHQ9 of 8 or greater. There was good correlation between the symptom severity assessments. We conclude that the tablet-based PHQ9 is an excellent and efficient method of screening for depression in attendees at primary health care clinics and that one in five people should be assessed further for depressive illness and possible intervention.

## 1. Introduction

Technology based assessments are increasingly implemented for medical assessment and monitoring of individuals with a variety of medical and psychiatric disorders. Weight management and diabetes monitoring programs use technological based programs effectively [1, 2]. Web-based assessments and monitoring programs have been in place to monitor psychiatric symptoms for several years [3–5]. Telephone based systems have demonstrated the efficacy of this technology in the field worldwide [6, 7], including Ecuador [8]. Tablet-based systems for capturing survey data are well established and used clinically as an extension of the medical data recording method [9]. Adapting technology based assessments and monitoring tools for psychiatric disorders is critical as it may improve efficiency; responses to standardized questions generate measures for clinical monitoring that are automated and accessible. The versatility of the computer tablet allows the technology to be adapted widely in primary care clinics. However, the use of computer tablets in an emerging health system, such as in Quito, Ecuador, has not been studied. The feasibility at the level of the patient population and the capacity to gather and record symptoms of depression are critical to successfully adapting a technology based screening and monitoring system for psychiatric symptoms in primary care.

The effective clinical management of major depression in primary care represents a significant opportunity for improvements in health, both in terms of the quality of individual life and the financial sustainability of community health delivery programs by enhanced adherence to medical interventions. Depression is a common disorder and is among the prominent and leading medical causes for disability worldwide [10, 11]. Depression is recognized as a major problem in the primary health care setting with a point prevalence between 5 and 10% and up to twice as many that experience depressive symptoms but do not rise to a full major depression diagnosis [12]. Individuals with depression have a threefold increase in comorbid chronic diseases, are less likely to seek and receive effective treatment for medical conditions, have lower rates of appropriate preventative health services and screening, and are less adherent to medical recommendations [13]. The majority of individuals with depression are treated by primary care providers who prescribe 80% of the antidepressant medications in the USA [14]. The burden of untreated depression at the primary care level is substantial as most depressions go unrecognized and untreated [15]. In addition to the personal and vocational cost of untreated depression, it has been shown that overall medical care costs are increased, with greater numbers of patient care provider interactions, investigations, and treatments for a variety of medical complaints [16]. Identification, diagnosis, and implementation of treatments specific for depression at the primary care level have substantial personal and medical implications for effective patient management in the community [17, 18].

Major depressive disorder is understudied in Ecuador. The few studies that address the prevalence of depressive disorders in Ecuador are limited to the public health sector collecting data on use of health services; by the year 2007 the rate of major depression was 72 per 100,000 inhabitants, low but a clear increase and effectively double the rate from the previous decade; by 2012 the rate of major depression was 162 per 100,000 [19]. Compared to the USA rates [20], these are lower rates; however, the increasing rates are likely to reflect an increased awareness of depression both at the care provider and patient focused levels. There is clear evidence of depressive disorders throughout the Ecuadorian society, including the indigenous populations [21, 22]. A study conducted in the rural and urban sectors of a Quito health area using the GHQ-12 found that 37.1% of the individuals surveyed had positive scores for mental health issues [23]. The global health perspective on the rates of depression throughout the world suggests that the prevalence is likely to be in the 10% range [24], significantly higher than estimated by public health records in the Ecuadorian government (1.6%) and consistent with observations elsewhere that up to 75% of depressions go undiagnosed and therefore untreated.

We tested the use of a computer tablet in the clinical setting to screen for depressive symptoms in a sample of attendees to a primary clinic program in Quito, Ecuador, in order to determine the feasibility and acceptance of the implementation of novel technology to assess depression symptoms by the patient population and compare two depressive symptom severity instruments, the 9-item Patient Health Questionnaire (PHQ9) [25] and the Quick Inventory of Depressive Symptoms-Self Report (QIDS-SR) [26], with the 12-item General Health Questionnaire (GHQ-12) [27]. We find that the technology was readily embraced and we find identified rates of depression in the attendees of a primary care clinic that were similar to those in the USA. We hypothesized that the clinic attendees would embrace the novel technology and that the screening questionnaires would identify individuals with depressive symptoms by increasing the efficiency of the clinical interaction.

## 2. Methods

### 2.1. Clinical Participants

Patients attending a primary health care clinic affiliated with Universidad San Francisco de Quito in Quito, Ecuador, were invited to participate anonymously in a series of self-administered questionnaires. Individuals were offered participation by the clinic staff at arrival and were evaluated prior to seeing the primary care physician (PCP). After informed consent was procured and the participants completed a short tutorial on the use of tablet (iPAD) in completing the questionnaires embedded in the “Polldaddy” survey software application, participants were asked to independently complete all three screening tools. The patients were screened by a team of medical students (DS, EF, and GJ) for depressive symptoms using a DSM-IV checklist for Major Depressive Disorder by the clinical researchers. Finally, the Clinical Global Impression-Severity (CGI-S) [28] rating by the primary health care provider recorded the clinician's impression of patient's level of depression from 1 (not at all depressed) to 7 (critically depressed). The CGI-S was completed after the survey questions were completed and after the individual had seen the PCP.

All clinical assessments were completed in Spanish. The instruments used were the nine-item Patient Health Questionnaire (PHQ-9) [29, 30], GHQ-12 (General Health Questionnaire) [27, 31], and QIDS-SR (Quick Inventory of Depressive Symptomology-Self Report) [26, 32]. The Spanish language versions of assessment instruments were uploaded to a central website (http://www.polldaddy.com) designed to host survey questionnaires that could be administered in the field using a computer tablet (iPad). The GHQ-12 was scored according to the method described by Goldberg et al. [27]. The PHQ-9 was scored according to the method described by Kroenke et al. [33]. The QIDS-SR was scored according to the method described by the Epidemiology Data Center of the University of Pittsburg (http://www.ids-qids.org/index2.html#SCORING).

Reliability was evaluated by comparing scores on the PHQ-9 with those on a measure reflecting depression symptomology previously studied in Latin American patients, the GHQ-12 [23]. Convergent and divergent validity was assessed with Spearman's Rho, comparing PHQ-9 scores to the scores of the GHQ-12 and QIDS-SR.

Feasibility was assessed quantitatively by recording the proportion of patients that were able to complete the questionnaires once they had agreed to participate. It was also qualitatively assessed through observations from the research assistants regarding ease of use among the different demographics that participated in the study.

## 3. Results

### 3.1. Descriptive Findings

This study engaged 226 participants between the ages of 18 and 65, attending a primary care clinic for nonpsychiatric reasons, that agreed to participate anonymously in a survey of depressive symptomatology using computer tablet-based technology. All 226 patients completed the PHQ-9, the GHQ-12, and the QIDS-SR. Based on the average number of daily contacts at the clinic, it is estimated that there were approximately 500–600 individuals that attended the clinic during the study time. A total of 131 CGI scores for these patients were collected as well; this proved to be somewhat more difficult to gather as it required the clinical researcher to interact with the primary care clinician, whose availability was often limited. The DSM-IV Major Depressive Episode checklist review was conducted on 188 patients (Table 1).

### 3.2. PHQ-9 Validity: Correlation of PHQ-9 Scores to other Depression Screening Tools

**Figure 1:**
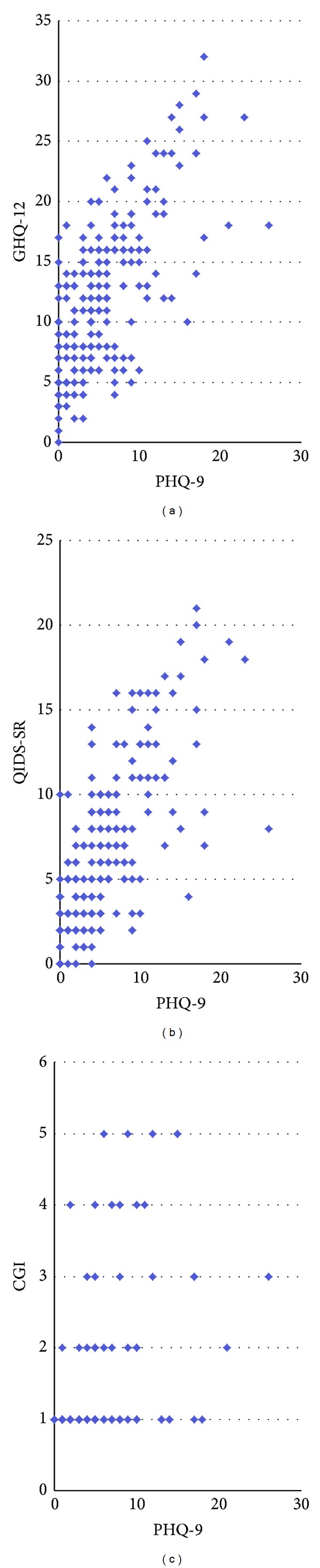
Depression severity scores of the PHQ9 and relationship to GHQ12, QIDS-SR, and CGI. There is an obvious correlation between GHQ12 and QIDS-SR with the PHQ9; the correlation is present with the CGI estimates; however, there are clearly a number of CGI estimates ≤3 that have PHQ9 scores suggestive of depression.

Table 2 presents the correlation coefficients between the PHQ-9 and the other depression assessment tools. The PHQ-9 results were compared to the data from the GHQ-12, the QIDS-SR, the DSM-IV, and the CGI. The PHQ-9 showed significant correlation with all measures: GHQ-12 (*r* = 0.64, *P* < 0.0001), QIDS-SR (*r* = 0.68, *P* < 0.0001), DSM-IV symptom count (*r* = 0.67, *P* < 0.0001), and CGI (*r* = 0.39, *P* < 0.0001).  Figure 1 further demonstrates the relationship between the PHQ measures with the QIDS-SR, GHQ-12, and CGI. Despite the apparent correlation, there was a broad range of CGI scores and 2/3 of individuals with CGI of 3 or less had a PHQ9 > 8, with the implication of depressive symptoms not suspected clinically.

### 3.3. Feasibility of Technology Use

All participants who started the survey (226/226) were able to complete the self-assessments on the iPad tablet computer. Five patients were unable to read the text on the tablet screen as a result of not bringing reading glasses to the doctor's office and were therefore excluded from participating.

## 4. Discussion

The integration of technology in the assessment and monitoring of depression represents a tangible advance in clinical care and increases efficiency in detection and treatment, particularly in communities with limited resources and capacity for medical care (device costs are decreasing). Screening for depression in a primary care setting of Quito, Ecuador, using computer tablet-based technology proved to be a very efficient and adaptable method of gathering information; it was readily accepted by the clinic attendees and used without difficulty following a brief orientation to the process by the researchers. A PHQ9 score of 8 was used as a threshold for screening purposes; it is well known that somatic symptoms are common (75%) in Latin American culture and there is a tendency to under represent mood symptoms [34]. In many primary care clinics, 75% of patients subsequently found to be depressed initiated the clinical visit because of somatic concerns, and depression was not the self-identified driving need for medical attention [35]. This argues for a lower threshold on the PHQ9 in cultures wherein psychological aspects of depression are likely to be less frequently acknowledged; we believe this will increase the sensitivity of the screening. There were instances initially wherein presbyopic participants were challenged and did not bring along reading glasses; this was solved by having off-the-shelf corrective reading glasses available; 4 patients who were unable to complete the forms are not included in the total 226 participants. All self-report questionnaires had been applied to psychiatric research and the construct validity of the measures has been established in the USA clinical setting [33, 36, 37]. In addition to piloting the use of technology at the clinical primary care level of Quito, it was our intent to evaluate the depressive symptom screening and severity instruments to estimate their clinical utility. We considered a control group with paper based screening; however, the project was designed to assess how well a technological approach could be adapted to an active clinic. We elected to use participation and completion of assessments as a measure of acceptability; in view of the fact that we were integrating into the clinic process and wished to gather data in an expedited manner, we elected to not gather information on the perspective of the participant on the process. While this may be considered a weakness of the study, we feel that the strength of the approach is the assumption that measurement based care is integral to health care at the primary level.

There was excellent correlation between the depression symptom severity assessment scores of the PHQ-9 and the QIDS-SR (0.68) which is expected as they are both designed to measure depression; however, this correlation is important as we are in fact measuring whether depression is identified with 2 separate measurement instruments. This was the case. There was excellent correlation with the number of DSM-IV symptoms acknowledged by the participant and their PHQ-9 scores, and, finally, there was good correlation with the GHQ scores, consistent with previous findings [29]. Studies of the PHQ9 in Latin America are few; however, a study in Honduras [38] indicated that it was a feasible measure of depression in this culture. The GHQ has been validated in Chile [39] as an efficacious measure of depressive symptomology, and has been used in Ecuador [23]. Thus, we conclude based on our correlated results that are consistent with strong convergent validity of the measures that they are valid measures of depressive symptoms in the Ecuadorian population.

The Clinical Global Impression-Severity (CGI-S) scale is a 7-point scale that is used to record the clinician's impression of the severity of the patient's illness at the time of assessment. It is a subjective scale and is generally reflective of the knowledge of the clinician about the patient and their illness. The correlation of CGI-S with depressive symptom ratings is generally good [40] and can be expected to predict symptomatic states. The observation that a substantial number of individuals with a CGI-S of >3 were present despite having relatively substantial symptoms of depression suggests that the clinicians who were asked to give their impression of the patient's depressive symptoms were unaware of the depression. This could be for a number of reasons; the patient may have attended the clinic for reasons not related to how they were feeling and did not disclose to the primary care physician that they were depressed. The PCP may not have asked about depressive symptoms and was unable to determine therefore if depression was present. The PCP may not have been attuned to the patient's medical complaints and was not concerned that the physical complaints could be driven in part by underlying depressive symptoms.

The health care system in Ecuador is state funded through the Ministry of Health; the first line of contact with the health care system is through the primary care physician who is responsible for the implementation of the government's health care policies. Ongoing efforts are focused on strategic implementation of preventative health screening in several dimensions such as diabetes and hypertension, but currently there are no policies for screening for depression or other psychiatric disorders. There is every advantage to a national screening program implemented systematically through the primary health care providers. There is considerable efficiency in screening for depression in the primary care setting [41, 42], particularly when available care is at hand. On the other hand, if there are no specific interventions to follow up on the screening process, there is questionable utility in screening at the population or primary care level for depression [43]. Since the management of depression is done by primary care providers in most health systems, it is most logical that the Ecuadorian health system engage their PCPs in the screening and management of depression. The paucity of published research in depression in the Latin American countries implies that they have similar challenges as Ecuador.

There are several advantages to screening depression and the use of electronic methods provides efficiency in data collection and management. The instruments used in the current study are easily adapted to electronic versions and showed high correlation between scores which suggests a consistency of measure of the condition (depression) that they were designed to assess. This is of importance at the level of primary care, where there frequently is no psychiatrist or mental health professional available to perform clinical evaluations. The importance of screening is reflected in the next steps, specifically what happens with the information and the subsequent actions. At what severity level does a symptom screen translate to a diagnosis of depression and a treatment plan? We find that 20% of individuals have a PHQ-9 > 8 and would recommend further evaluation at the primary care level for major depression and possible treatment. Given the level of comorbid medical chronicity and other potential personal, medical, and social consequences of depression, the combined economical and health benefits of treatment of depression are clear. There is evidence that medical assistants are effective in follow-up of screening assessments and facilitators of treatment and intervention strategies [44], providing practical assessment to the primary care team before the referral for specialty evaluation. The strategies of an electronically based screening process such as that performed in this project are an effective first step in the identification and management of depression.

The limitations of this report include the lack of a thorough follow-up examination with a detailed clinical interview to verify the diagnosis. Further, this was not a systematic screening of attendees and so a rate of depression cannot be established. The primary purpose of this work was to assess the efficiency of the tablet-based screening in the clinics.

We find that one in five individuals attending a primary health care clinic reports a PHQ-9 > 8 and of those individuals only a small percentage had CGI-S scores > 3 suggesting that concerns were noted by the clinician infrequently. The cut-off of 8–10 for the PHQ-9 has been suggested in populations with somatic complaints [45]. This is consistent with the observation in the literature that approximately 50% of depressions are missed in primary care. We were able to gather CGI-S in only half of our participants, as the availability of the PCP was limited, which again reflects the busy nature of a primary health care clinic.

Depression is frequently overlooked in the clinical setting in Ecuador and this is reflected in the low rates of depression reported by the Ministry of Public Health. The recent increases in the reported rates of depression [19] suggest that there is an increasing awareness to this disease; however, the rates are still substantially lower than elsewhere and there is every likelihood that the Ecuadorian rates parallel those elsewhere [24]. Primary care is clearly the clinical contact point for individuals to be screened for depression and use of computer technology in the screening process is demonstrated to be very effective and acceptable to the population. The PHQ-9 and the shorter PHQ-2 are excellent methods for screening for depressive symptoms to be followed up with a more detailed assessment and treatment.

Screening for depression is only one step in the overall management of depression; clearly it is necessary to detect and diagnose the illness in order to effectively treat it. However, merely detecting depression at the primary care level in the absence of a program to manage the disorder is of minimal consequence [46, 47]. Detecting elevated blood glucose is of no use unless a system is in place to diagnose and treat diabetes. There are many options available following a positive screening finding for depression. They range from a diagnostic interview by a clinician with experience in mental health to determine the nature of the problems and the specific needs of the patient to education programs on health and illness management. The treatment plan is subsequently tailored to the needs of the patient and the capacity of the care providers. Critical to the management is a systematic method of screening and monitoring of symptoms and we have demonstrated that a tablet-based system is an effective and acceptable method that is easy to use in any clinic setting.

## Figures and Tables

**Table 1:** 9-item patient health questionnaire (PHQ9), 12-item general health questionnaire (GHQ12), quick inventory of depressive symptoms-self-report (QIDS-SR), clinical global impression (CGI), and DSM-IV symptom counts description of total number of assessments, mean, and range of scores.

Assessment Tool	*n*	Mean (SD)	Range
PHQ9	217	5.41 (4.99)	0–26
GHQ12	226	11.93 (6.12)	0–32
QIDS-SR	226	6.36 (4.53)	0–21
CGI	128	1.51 (1.06)	1–5
DSMIV symptom count	188	1.50 (2.00)	0–8

**Table 2:** Correlation between tests scores.

	PHQ-9	GHQ-12	QIDS-SR	CGI
GHQ-12	0.63***			
QIDS-SR	0.68***	0.57***		
CGI	0.44***	0.36***	0.34***	
DSM-IV SX count	0.72***	0.60***	0.73***	0.48***

***P value < 0.001.
